# Texas Students

**Published:** 1893-04

**Authors:** 


IT is always a source of pleasure and satisfaction to the TEXAS MEDICAL JOURNAL to see Texas students acquit themselves with credit at commencement day, thus upholding the credit of the State, and the reputation they have for winning honors in the front ranks.

At the recent commencement of the Medical Department of the University of Tennessee, in a graduating class of one hundred and fourteen, there were fifteen Texas students; and the honor of delivering the valedictory fell upon a Texan, Dr. PHILIP RUSSELL SIMMONS, of Sipe Springs Texas. The *Southern Practitioner* says "he acquitted himself of the difficult task with honor, and delivered the farewell gracefully, indulging often in eloquent metaphorical flights." Prof. Cain in a private letter to the *Journal*, also bears testimony to the creditable manner in which Dr. S. acquitted himself; and of other Texas students graduated, Dr. Cain speaks as follows:

"There were two other Texas students who were honored during the past session. Vacancies in the interne-ships of two of our hospitals, St. Margarets' and the hospital of the Good Shepherd, occurred early in the session and it became necessary to fill them from our class. Dr. JOHN M. STRAYHORN, of Bartlett Texas, and DR. JERRY ASHLEY, of Pecan Gap, both under graduates [at the time] but accomplished physicians, were placed in the positions, and discharged well the duties."

"The *Southern Practitioner* says: The class was the largest ever graduated from even this popular and worthy institution, and goes to show that the fame of the able Faculty which conducts it has gone abroad, and is attracting attention of young men wishing to study medicine, all over the country."

Dr. Strayhorn visited Austin since his return from Nashville, and joined the Austin District Medical Society, and the next thing he did was to subscribe for his *home journal.*

